# AMPK Mediates Glucocorticoids Stress-Induced Downregulation of the Glucocorticoid Receptor in Cultured Rat Prefrontal Cortical Astrocytes

**DOI:** 10.1371/journal.pone.0159513

**Published:** 2016-08-11

**Authors:** Shi-Ying Yuan, Jue Liu, Jun Zhou, Wei Lu, Hai-Yun Zhou, Li-Hong Long, Zhuang-Li Hu, Lan Ni, Yi Wang, Jian-Guo Chen, Fang Wang

**Affiliations:** 1 Department of Pharmacology, School of Basic Medicine, Tongji Medical College, Huazhong University of Science and Technology, Wuhan, Hubei, China; 2 Department of Pharmacy, The Central Hospital of Wuhan, Tongji Medical College, Huazhong University of Science and Technology, Wuhan, China; 3 Key Laboratory of Neurological Diseases (HUST), Ministry of Education of China, Wuhan, Hubei, China; 4 The Key Laboratory for Drug Target Researches and Pharmacodynamic Evaluation of Hubei Province, Wuhan, China; 5 The Institute of Brain Research, Huazhong University of Science and Technology, Wuhan, China; Université catholique de Louvain, BELGIUM

## Abstract

Chronic stress induces altered energy metabolism and plays important roles in the etiology of depression, in which the glucocorticoid negative feedback is disrupted due to imbalanced glucocorticoid receptor (GR) functions. The mechanism underlying the dysregulation of GR by chronic stress remains elusive. In this study, we investigated the role of AMP-activated protein kinase (AMPK), the key enzyme regulating cellular energy metabolism, and related signaling pathways in chronic stress-induced GR dysregulation. In cultured rat cortical astrocytes, glucocorticoid treatment decreased the level, which was accompanied by the decreased expression of liver kinase B1 (LKB1) and reduced phosphorylation of AMPK. Glucocorticoid-induced effects were attenuated by glucocorticoid-inducible kinase 1 (SGK1) inhibitor GSK650394, which also inhibited glucocorticoid induced phosphorylation of Forkhead box O3a (FOXO3a). Furthermore, glucocorticoid-induced down-regulation of GR was mimicked by the inhibition of AMPK and abolished by the AMPK activators or the histone deacetylase 5 (HDAC5) inhibitors. In line with the role of AMPK in GR expression, AMPK activator metformin reversed glucocorticoid-induced reduction of AMPK phosphorylation and GR expression as well as behavioral alteration of rats. Taken together, these results suggest that chronic stress activates SGK1 and suppresses the expression of LKB1 via inhibitory phosphorylation of FOXO3a. Downregulated LKB1 contributes to reduced activation of AMPK, leading to the dephosphorylation of HDAC5 and the suppression of transcription of GR.

## Introduction

Chronic stress plays an important role in the pathogenesis of stress-related psychiatric diseases, such as depressive disorders [1]. Under normal conditions, stress exposure leads to the activation of the hypothalamus-pituitary-adrenal (HPA) axis and the elevation of glucocorticoids (GCs), which regulates the activity of HPA axis through a negative-feedback involving the glucocorticoid receptor (GR) in the brain [2]. However, prolonged exposure to stress alters the function and expression of GR resulting in defective glucocorticoid negative feedback [3]. An extensive body of literature reports that corticosterone (CORT) administration induces reliable and robust depression-like behaviors in animal models [4, 5].

Recent studies have suggested that the intracellular energy metabolism may explain the depressive behaviors induced by chronic stress [6–8]. AMP-activated protein kinase (AMPK) is an enzyme which plays a key role in cellular energy metabolism [9]. An increased AMP to ATP ratio leads to activation of AMPK by its upstream kinases, such as AMPK kinase, liver kinase B1 (LKB1) and calmodulin-dependent kinases, which phosphorylate threonine 172 of the α-subunit. In addition, allosteric activation and inhibition of dephosphorylation by protein phosphatases also contribute to the activation of AMPK [10]. In the central nervous system (CNS), AMPK participates in fasting, inflammation, stress and other responses [11–14]. Decreased phosphorylation and inactivation of AMPK has been shown to be associated with depression-like behaviors in rats and mice exposed to chronic stress [7, 8]. These studies suggest that AMPK may play an important role in stress-induced behavioral changes or psychiatric disorders. In the periphery, interestingly, depending on the tissue in the periphery, the regulatory function of GCs appears to be different [15]. For instance, GCs decrease the activity of AMPK in the adipose tissue and heart, while it promotes AMPK activation in the liver and hypothalamus [16]. In addition, AMPK can regulate GR function through p38 MAPK pathway [17]. However, the relationship between GCs and AMPK in the CNS needs further elucidation.

Astrocytes, the most numerous cell type in the brain, are an important source of ATP and neurotrophins (NTFs), which maintain the normal function of neurons [6, 18]. Recent studies demonstrate that astrocytes play important roles in neuropsychiatric disorders, such as major depression and schizophrenia [19, 20]. The loss of astrocytes was observed in the cerebral cortex of patients with major depressive disorders (MDD) [21]. Reduced expression of GR after chronic exposure to GCs has been shown to account for the loss of astrocytes [22]. GR in astrocytes, as a critical stress-responding transcriptional factor, may mediate stress-induced adaptation via modulating the expression of astrocyte-derived NTFs.

Nevertheless, to the best of our knowledge, the association between AMPK and GR in the condition of chronic exposure of GCs in astrocytes is unclear. In the current study, we investigated the role of AMPK in GCs stress-induced down-regulation of GR in rat astrocytes. Our findings identify AMPK as an integral component involved in the maintenance of GR function in normal and stress conditions. GCs stress activates glucocorticoid-inducible kinase 1 (SGK1) and inhibits AMPK activation via Forkhead box O3a (FOXO3a)-mediated downregulation of LKB1. The inactivation of AMPK promotes the activation of histone deacetylase 5 (HDAC5) resulting in decreased expression of GR after chronic exposure to GCs. In line with these, the activation of AMPK reverses GCs stress-induced depressive behavior and GR down-regulation.

## Materials and Methods

### Animals

All animal care and experimental procedures were complied with the Guide for Care and Use of Laboratory Animals as adopted and promulgated by the National Institutes of Health. The use of animals for all experimental procedures was also approved by the Animal Welfare Committee of Huazhong University of Science & Technology. The behavioral experiments were conducted with 4- to 5-month-old male Sprague-Dawley (SD) rats. Up arrival, rats were maintained on a controlled 12:12 light-dark cycle at a constant temperature (22 ± 2°C) with ad libitum access to food and water. Rats were weighted and monitored every day carefully to ensure all rats behave normally. There was no abnormal casualty in whole process. Animals were sacrificed by decapitation following anesthesia with isoflurane. Other efforts were made to minimize suffering and to reduce the number of animals used in the experiments.

### Agents

Corticosterone (CORT), dexamethasone (DEX), Compound C, 9-β-D-arabinofuranoside (Ara-A), 5-aminoimidazole-4-carboxamide-1-β-D-ribofuranoside (AICAR), aspirin, metformin, fluoxetine, RU38486, GSK650394, SAHA, SB939, Hoechst33258 and poly-L-lysine were purchased from Sigma-Aldrich (St. Louis, MO, USA). Anti-AMPK, anti-p-AMPK (Thr172), anti-GR, anti-FOXO3a, anti-p-FOXO3a (Thr32), anti-LKB1 and anti-HDAC5 antibodies were obtained from Cell Signaling Technology (Boston, MA, USA). Anti-p-HDAC5 (S259) and anti-p-HDAC5 (S498) antibodies were purchased from Abcam (Cambridge, MA, USA). Anti-GAPDH and anti-GFAP (glial fibrillary acidic protein) antibodies were purchased from Santa Cruz (Dallas, Texas, USA). Dulbecco’s modified Eagle’s medium DMEM/F12 was obtained from Biotium (San Francisco, CA, USA) and Gibco Invitrogen Corporation (Carlsbad, CA, USA). Heat-inactivated fetal bovine serum was purchased from Hyclone (Logan, UT, USA). Other agents were purchased from commercial suppliers. All drugs were prepared as stock solutions and stored at -20°C. The final concentration of DMSO was less than 0.05%. No detectable effect of DMSO was found in all the experiments. The final concentrations of drugs were determined according to clinical usage or physiological levels.

### Cell culture

Newborn SD rats (day 1–3) were obtained from the Experimental Animal Center of Tongji Medical College, Huazhong University of Science & Technology. The procedure was conducted according to the Guide for Care and Use of Laboratory Animals. Prefrontal cortical astrocytes were prepared as previously described [23]. In brief, the cerebral cortices of postnatal day 1–3 SD rats were collected and incubated with 0.125% trypsin for 20 min at 37°C. After centrifugation at 1000 g for 6 minthe cells were resuspended and plated on culture flasks coated with poly-L-lysine (1 mg/mL). Then cultures were incubated in DMEM/F12 supplemented with 10% heat-inactivated fetal bovine serum, L-glutamine (2 mM) and 1% penicillin-streptomycin (100 U/mL) at 37°C in a humidified 5% CO_2_ atmosphere. Then, the medium was changed to DMEM/F12 after 24 h, and replaced every 3 days. After 9 days, the cells became confluent, non-astrocyte cells were removed by shaking (37°C, 150 g/min, 15 h). The astrocytes were used for experiments 12–14 days after plating. Immunofluorescence with glial fibrillary acidic protein (GFAP) antibody was used to identify the astrocytes. The purity of astrocytes was more than 95%.

### Immunofluorescence

For immunofluorescent assay, the cultured astrocytes were fixed with 4% paraformaldehyde in 10 mM phosphate-buffered saline (PBS; pH 7.4) for 20 min at room temperature, blocked with 3% normal goat serum (Sigma-Aldrich) for 30 min at room temperature, and incubated with rabbit anti-GR antibody (1:100, monoclonal, Cell Signaling, Boston, MA, USA) and mouse anti-GFAP antibody (1:200, monoclonal, Abcam, Cambridge, UK) overnight at 4°C. Then, the cells were washed with PBS, incubated with TRITC-conjugated goat anti-rabbit IgG (1:200; Vector, Burlingame, CA, USA) and FITC-conjugated goat anti-mouse IgG (1:200; Vector) secondary antibodies for 60 min while shaking at room temperature in darkness, and washed with PBS. Finally, the astrocytes were incubated for 2 min with nucleic acid stain, Hoechst33258 (Invitrogen, Carlsbad, CA, USA), diluted 1:5000 in PBS. All primary and secondary antibodies were diluted in PBS containing 1% normal goat serum. The images were captured using a confocal laser scanning microscopy (LSM 710, Carl Zeiss, Germany).

### Western blotting

The procedure of western blot analysis was processed according to the previous study [24]. Briefly, after boiled in SDS sample buffer (5 min), equal amounts of protein were loaded in each lane and separated on 10% SDS/PAGE gels. Samples containing 20 μg proteins were separated electrophoretically and then transferred to nitrocellulose membranes by using a transferring system (Bio-Rad, California, USA). After blocking with 5% nonfat dried milk powder/TBS/0.1% Tween 20 (1 h at room temperature), membranes were probed with the appropriate antibodies overnight at 4°C. Membrane-bound primary antibodies were detected using secondary antibodies conjugated with horseradish peroxidase (1:4000) (Zhongshan Biotechnology Company, Beijing). Immunoblots were developed on Micro Chemi (DNR Bio-imaging systems, Jerusalem, Israel) using the enhanced chemiluminescence technique (ECL; Pierce Chemical Co., Rockford, IL, USA). All assays were performed at least three times. The optical densities of the detected bands were quantified using Scion Image software (Fredrick, MD, USA). Results were presented as percentage of control after normalization.

### RNA interference

A third generation of self-inactivating lentivirus vector (GeneChem, Shanghai, China) containing a CMV-driven GFP reporter and a U6 promoter upstream of the cloning sites (Age I and EcoR I) was used for cloning small hairpin RNAs (shRNAs). The targeting sequence for rat AMPKα2 gene (GeneBank NM_023991) was designed as follows: 5’-GCTGACTTCGGACTCTCTA-3’. The negative control sequence was 5’-TTCTCCGAACGTGTCACGT-3’, which was a randomly chosen nonsense sequence. The cultured astrocytes 10 days after plating were infected with lentivirus at a multiplicity of infection (MOI) of 20 for 8 h. Then, the medium was replaced with fresh complete medium. After 3 days, cells were observed under fluorescence microscopy to confirm that more than 80% of cells were GFP-positive, which means successful infection of the lentivirus and expression of the cloning sequences.

### Chronic GC exposure

Chronic glucocorticoids stress was mimicked by chronic exposure to CORT [4]. Rats were divided randomly into four groups (vehicle, CORT, CORT + fluoxetine and CORT + metformin). CORT or vehicle was given via subcutaneous (s.c.) injection at 9:00 am to 11:00 am every day for 21 days, which was similar to the endogenous release of glucocorticoids. From the 15th day, metformin (Met) or fluoxetine (Flu) was given via intragastric administration for 14 days. During the 28 days of drug administration, the weight of each rat was measured every day.

### Forced swimming test

Twenty-four hours after the final administration of drugs, forced swimming test (FST) was carried out to examine the depression level of the rats. As described in published literature [25], the apparatus used for FST of rats was placed in a quiet behavioral experiment room, with a water depth of 35 cm and water temperature at 23–25°C. Rats were put into the swimming apparatus individually and adapted for 2 min. Non-swimming time (immobility time) was subsequently recorded for 4 min. Animals were removed from the forced swim test tank when the mouth and nose of rat was submerged below the surface of the water for 5 seconds. The whole process was recorded by a camera. The criterion for immobilization was vertical floating in the water and movements necessary to keep the head above the water. Every rat was wiped dry immediately after the test and the apparatus was cleaned to avoid cross influence on the next rat. The immobility time was used as an indicate evaluating the stress behavior of rats. No animal died or injured in the whole process.

### Statistical analysis

Data are normalized to control and presented as mean ± SEM. Statistical analysis was performed using SPSS 18.0 software (SPSS Inc., USA). Comparisons of the means between three or more independent groups were carried out using one-way ANOVA followed by LSD (least significant difference) tests. P < 0.05 was considered statistically significant.

## Results

### Glucocorticoids decreases the expression of GR and AMPK activation in cultured rat prefrontal cortical astrocytes

**Fig 1:**
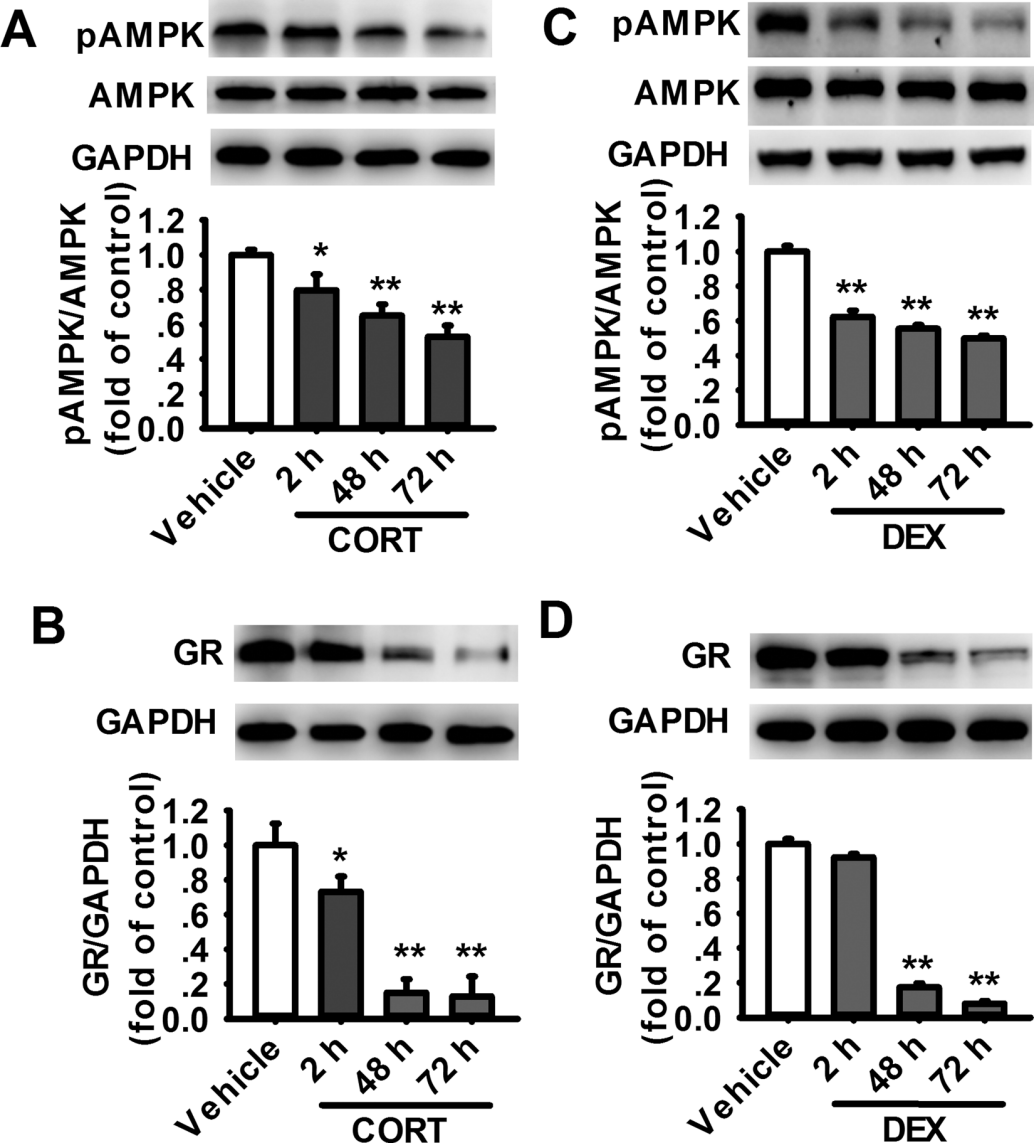
Exposure to glucocorticoids decreases the levels of pAMPK and GR in cultured rat prefrontal cortical astrocytes. Representative western blot (upper) and summarized data (lower) showing the level of pAMPK in cultured astrocytes exposed to CORT (A) or DEX (C) for 2 h, 48 h and 72 h. (n = 5 for each group). Representative western blot and summarized data showing the expression of GR in cultured astrocytes exposed to CORT (B) or DEX (D) for 2 h, 48 h and 72 h. (n = 4 for each group treated with CORT and n = 6 for each group treated with DEX). *P < 0.05, **P < 0.01 vs vehicle.

To examine the effects of GCs on astrocytes, both endogenous CORT (1 μM) and long-acting semisynthetic glucocorticoid dexamethasone (DEX, 1 μM) were administrated for 2 h, 48 h or 72 h on the cultured prefrontal cortical astrocytes to imitate the effects of stress-induced glucocorticoids release. Data presented in Fig 1 showed that the expression of both phosphorylated AMPK (pAMPK) and GR were significantly decreased after the treatment with CORT (Fig 1A and 1B) or DEX (Fig 1C and 1D). It was further shown that the inhibitory effects of CORT and DEX increased gradually with the rise of incubation time during a 72-h period. Consistent with the recent report [8], the expression of total AMPK was not changed after treatment with glucocorticoids (data not shown). To further examine whether the effects of GCs on the expression of GR and AMPK are specific for astrocytes, we performed western blot on alone cultured cortical neurons exposed to GCs chronically. The results suggested that there were no significant effects on the level of pAMPK or the expression of GR in cultured cortical neurons exposed to GC chronically (S1 Fig). The effects of chronic GCs exposure maybe mainly occurred in astrocytes, while not neurons.

### Inhibition of AMPK activity suppresses the expression of GR in cultured rat cortical astrocytes

**Fig 2:**
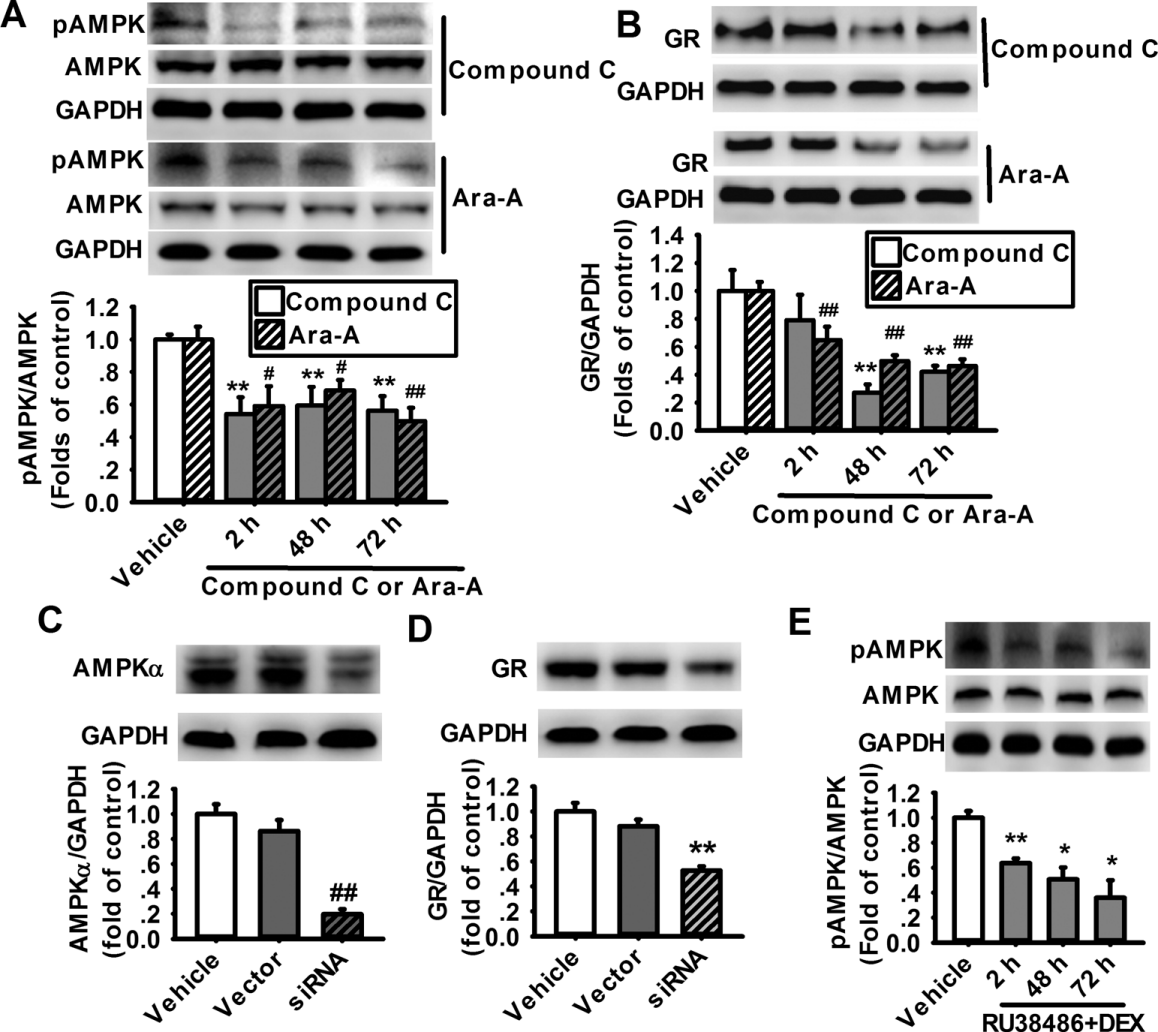
Inhibition of AMPK reduces the expression of GR in cultured prefrontal cortical astrocytes. Western blot images (upper) and summarized data (lower) showing the phosphorylation of AMPK (A) and GR expression (B) in the cultured prefrontal cortical astrocytes treated with 10 μM Compound C (n = 5–6 for each group) or 500 μM Ara-A (n = 7 for each group) for 2 h, 48 h and 72 h. **P < 0.01 vs vehicle (for treatment with Compound C). ^#^P < 0.05, ^##^P < 0.01 vs vehicle (for treatment with Ara-A). Representative western blot images and summarized data showing the expression of AMPKα (C) and GR (D) in the cultured astrocytes treated with AMPKα-siRNA for 12 h. (n = 6 for each group). ^##^P < 0.01 vs vector. (E) Representative western blot images and summarized data showing the level of pAMPK in the cultured astrocytes treated with 1 μM DEX and 3 μM RU38486 for 2 h, 48 h and 72 h. (n = 4 for each group). *P < 0.05, **P < 0.01 vs vehicle.

Since the activation of AMPK is closely related to environmental factors and the expression of many immediate-early genes, including transcriptional factors under the condition of stresses [26–28], we asked whether the suppression of AMPK activity mediates the decrease of GR expression induced by DEX in cultured prefrontal cortical astrocytes. Two commonly used AMPK inhibitors, Compound C (10 μM) and Ara-A (500 μM) were administrated respectively on the cultured astrocytes for 2 h, 48 h and 72 h. After treatment with Compound C or Ara-A, the level of pAMPK and the expression of GR were successfully suppressed compared with the control (Fig 2A and 2B).

To further confirm the effects of AMPK on the expression of GR, we knocked down AMPKα with specific small-interfering RNA (siRNA) targeting AMPK alpha 2 subunit. The cultured astrocytes were infected with vector lenti-viruses carrying the AMPKα-siRNA or the control siRNA. The results showed that the expression of AMPK in AMPKα-siRNA treated group was significantly lower than that in the vector-infected group (Fig 2C). Interestingly, the expression of GR was also significantly reduced in the AMPKα-sliencing group when compared with the vector group (Fig 2D).

In order to identify whether glucocorticoids-induced downregulation of GR and pAMPK is mediated by the GR itself in astrocytes, RU38486 (3 μM), a pharmacological blocker of GR [29], was administrated together with DEX for 2 h, 48 h and 72 h. The result showed that RU38486 could not prevent the DEX-induced suppression of AMPK (Fig 2E). These results suggest that AMPK plays a crucial role in the regulation of GR expression. However, the decreased activity of AMPK induced by GCs is independent of GR.

### Activation of AMPK reverses DEX-induced reduction of GR expression

To further testify the effect of AMPK on GR expression in prefrontal cortical astrocytes, we examined the effects of AMPK activators on DEX-induced downregulation of GR. We had previously observed that the effect of AICAR on pAMPK was up to maximum at 24 h, therefore, after incubation with DEX for 48 h, the astrocytes were further treated with AMPK activators (AICAR, 1 mM; aspirin, 5 mM or metformin, 2 mM) for 24 h respectively. The levels of pAMPK and GR were measured using western blot assay.

**Fig 3:**
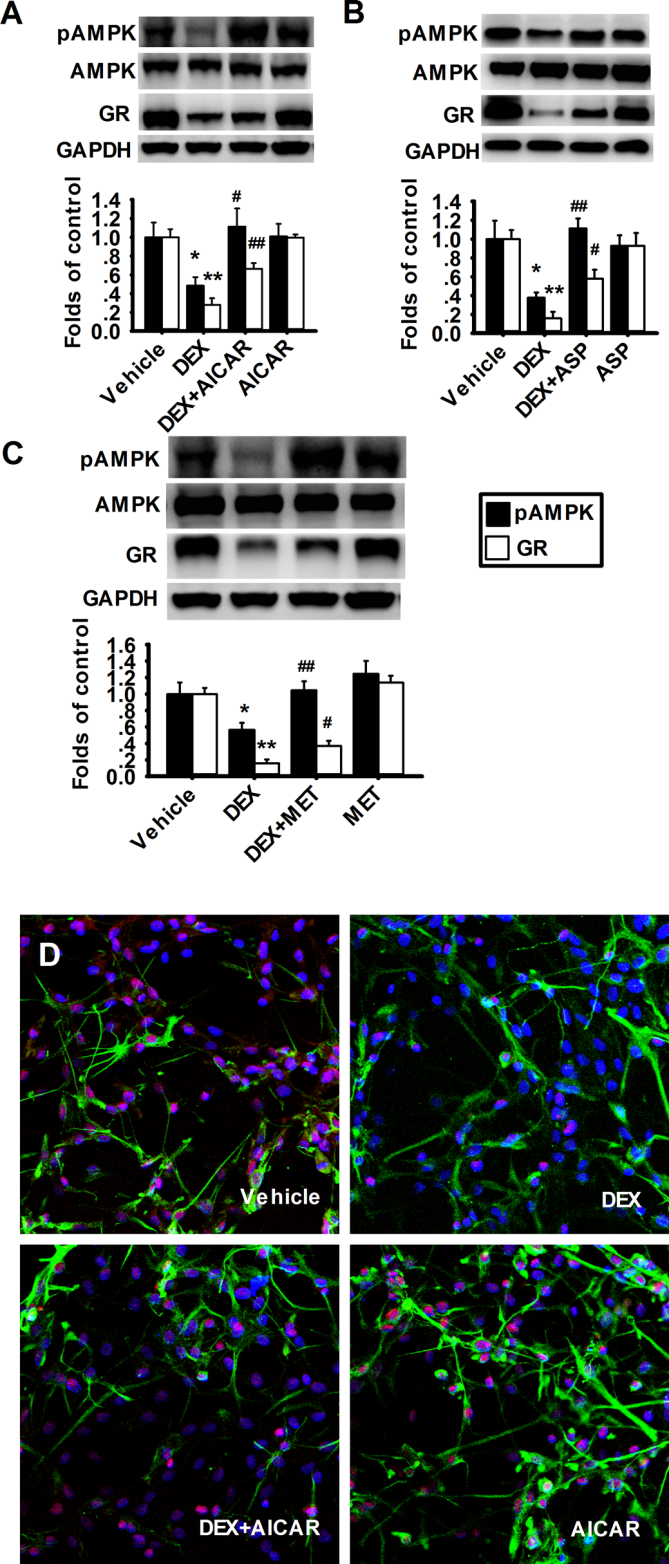
AMPK activators reverse the decreased expression of GR induced by DEX in cultured prefrontal cortical astrocytes. (A) Representative western blot images (upper) and summarized data (lower) showing the effects of AICAR (n = 5 for each group) (A), ASP (n = 4 for each group) (B), and MET (n = 7 for each group) (C) on DEX-induced decreases of GR and pAMPK in cultured astrocytes. (D) Immunofluorescent images of the cultured prefrontal cortical astrocytes treated with vehicle, DEX, DEX+AICAR or AICAR showing different localization of GFAP (green, cell body and apophysis of astrocyte), Hoechst33258 (dark blue, nucleus) and GR (pinkish red). Treatment with 1 mM AICAR for 24 h obviously increased the level of GR when compared with DEX group. *P < 0.05, **P < 0.01 vs vehicle. ^#^P < 0.05, ^##^P < 0.01 vs DEX.

In the astrocytes treated with AICAR and DEX, the levels of pAMPK and GR were both higher than those in the DEX-treated group (Fig 3A). Furthermore, the results from immunofluorescent assay showed that DEX decreased the expression and nuclear localization of GR. Similarly, the other two AMPK activators, aspirin and metformin also prevented the decrease of pAMPK and GR in prefrontal cortical astrocytes induced by DEX (Fig 3B and 3C). These alterations could be partially restored by AICAR (Fig 3D). The results suggest that AMPK activators can reverse the DEX-induced decrease in GR expression in cultured prefrontal cortical astrocytes, which further indicates that the suppression of AMPK activity mediates the reduction of GR expression in chronic glucocorticoids stress.

### Activation of SGK1 and inhibition of FOXO3/LKB1/AMPK signaling mediate DEX-induced decrease of GR expression

**Fig 4:**
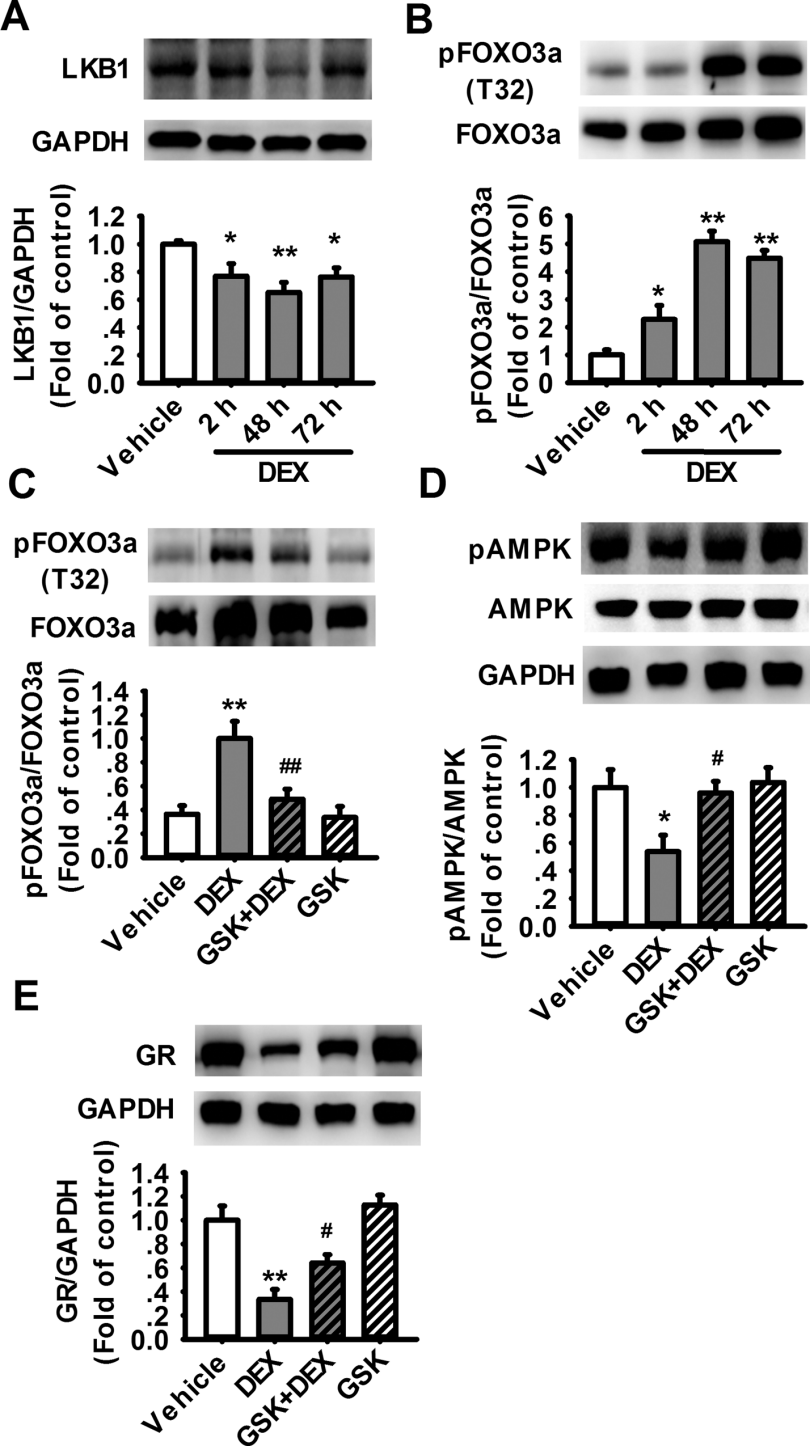
FOXO3a mediates the downregulation of GR induced by chronic exposure to GCs. (A) Representative western blot images and summarized data showing the decreased expression of LKB1 (A) and the inhibitory phosphorylation of FOXO3a-Thr32 (B) in cultured astrocytes (n = 6 for each group). (C) Representative western blot images and summarized data showed that GSK650394 prevented the increase in FOXO3a-Thr32 phosphorylation induced by DEX in cultured astrocytes (n = 6 for each group). Representative western blot images and summarized data showed that GSK650394 prevented the decrease in AMPK-Thr172 phosphorylation (n = 5 for each group) (D) and the decrease in GR expression (n = 4 for each group) (E) induced by DEX in cultured astrocytes. *P < 0.05, **P < 0.01 vs vehicle. ^#^P < 0.05, ^##^P < 0.01 vs DEX.

It has been demonstrated that glucocorticoid-inducible kinase 1 (SGK1) can be activated by GCs and stress, which further inactivates FOXO3a by inhibitory phosphorylation at the FOXO3a-Thr32 residue, thereby inhibiting the transcriptional function of FOXO3a [30, 31]. Liver kinase B1 (LKB1) is an upstream kinase of AMPK, and the transcription of LKB1 is regulated by FOXO3a [31]. Considering that chronic exposure to GCs decreased the level of pAMPK, we then examined the effects of DEX on LKB1 and FOXO3a at first. Our results showed that DEX exposure reduced the expression of LKB1 (Fig 4A) and increased the inhibitory phosphorylation of FOXO3a (Fig 4B). Next we examined the role of SGK1 in DEX-induced alterations of pFOXO3a and pAMPK using SGK1 inhibitor GSK650394 (GSK). It was shown that GSK (400 nM) significantly attenuated DEX-induced inhibitory phosphorylation of FOXO3a (Fig 4C) and prevented DEX-induced decrease in pAMPK (Fig 4D) and GR (Fig 4E). Consistent with the previous work, our results showed that SGK1/FOXO3/LKB1 signaling may contribute to the decrease in AMPK activation induced by chronic exposure to GCs.

### Dephosphorylation of HDAC5 mediates chronic DEX-induced decrease in GR expression in cultured astrocytes

**Fig 5:**
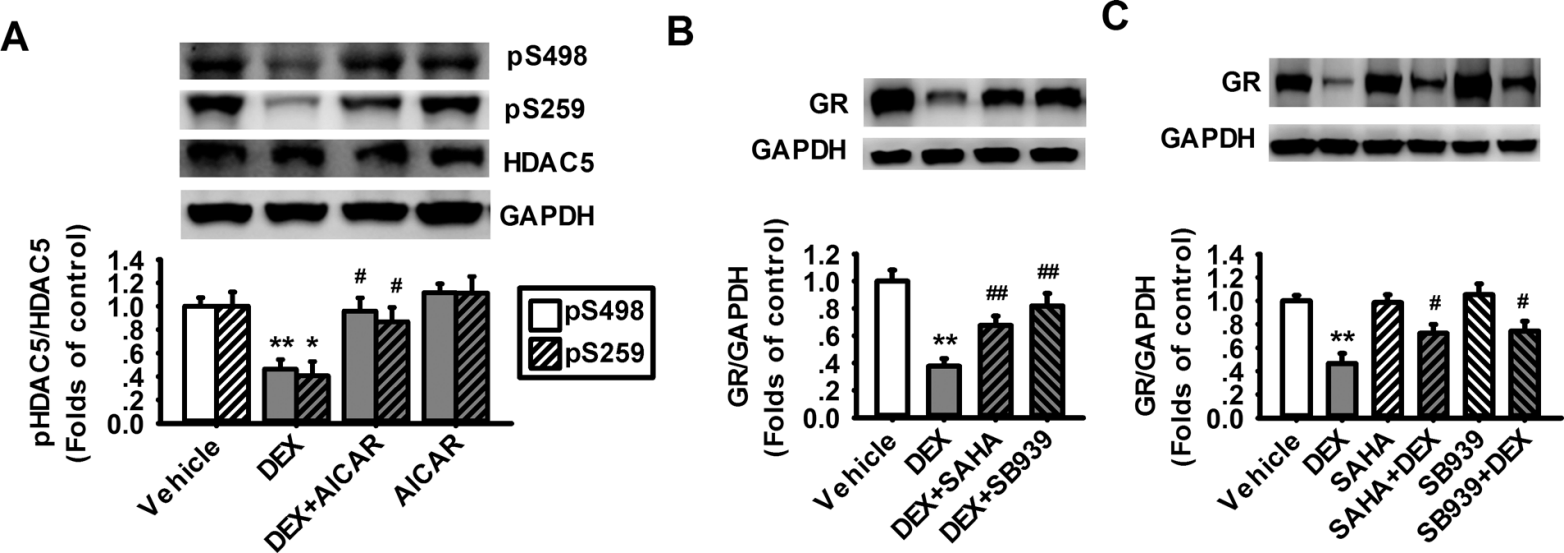
HDAC5 inhibitors reverse the decreased expression of GR induced by DEX in cultured prefrontal cortical astrocytes. (A) Representative western blot images and summarized data showing the phosphorylations (Ser498 and Ser259) of HDAC5 in the cultured astrocytes treated with 1 μM DEX alone for 48 h (DEX), 1 μM DEX for 48 h and then 1 mM AICAR for 24 h (DEX+AICAR), and 1 mM AICAR alone for 24 h (AICAR). (n = 4 for each group). (B) Representative western blot images and summarized data showing the level of GR expression in cultured astrocytes treated with 1 μM DEX for 48 h (DEX), 1 μM DEX and 500 nM SAHA together for 48 h (SAHA+DEX), and 1 μM DEX and 1 μM SB939 together for 48 h (SB939+DEX). (n = 8 for each group). (C) Representative western blot images and summarized data showing the level of GR in cultured astrocytes treated with 1 μM DEX for 48 h (DEX), 1 μM DEX for 48 h and then 500 nM SAHA for 24 h (DEX+SAHA), and 1 μM DEX for 48 h and then 1 μM SB939 for 24 h (DEX+SB939). (n = 8 for each group). *P < 0.05, **P < 0.01 vs vehicle. ^#^P < 0.05, ^##^P < 0.01 vs DEX.

AMPK has been reported to suppress the activity and nuclear translocation of histone deacetylase 5 (HDAC5) via phosphorylation of HDAC5 at the Ser259 and Ser498 sites. We investigated whether HDAC5 played a role in AMPK-mediated regulation of GR expression in prefrontal cortical astrocytes by employing HDAC5 inhibitors SAHA (1 μM) and SB939 (500 nM). The cultured astrocytes were treated with DEX (1 μM) for 48 h and then treated with AICAR, SAHA or SB939 for 24 h. As shown in Fig 5A, the phosphorylation of HDAC5 at Ser259 and Ser498 sites were both reduced by DEX treatment. Furthermore, the AMPK activator AICAR prevented the decreased of phosphorylated HDAC5 induced by DEX. These results suggest that chronic exposure to DEX decreases the phosphorylation of HDAC5, which could be restored by AMPK activators.

To investigate the effect of HDAC5 in the expression of GR, the cultured astrocytes were treated with DEX in the absence or presence of HDAC5 inhibitors SAHA or SB939 for 24 h (DEX+SAHA/SB939). We found that the expression of GR in DEX+SAHA and DEX+SB939 groups was largely rescued when compared with the DEX alone group (Fig 5B). This result suggests that inhibiting the activity of HDAC5 reverses DEX-induced decrease in GR expression. Furthermore, we treated the cultured astrocytes with DEX and HDAC5 inhibitors together for 48 h (SAHA/SB939+DEX). The results showed that the expression of GR was significantly higher in the SAHA/SB939+DEX groups than that in the DEX alone group (Fig 5C). This result demonstrates that inhibiting HDAC5 can prevent DEX-induced downregulation of GR. Together, these results indicate that increased activity of HDAC5 mediates the decreased expression of GR induced by DEX.

### Metformin reverses chronic CORT exposure-induced behavioral alteration and downregulation of GR in prefrontal cortex of rats

**Fig 6:**
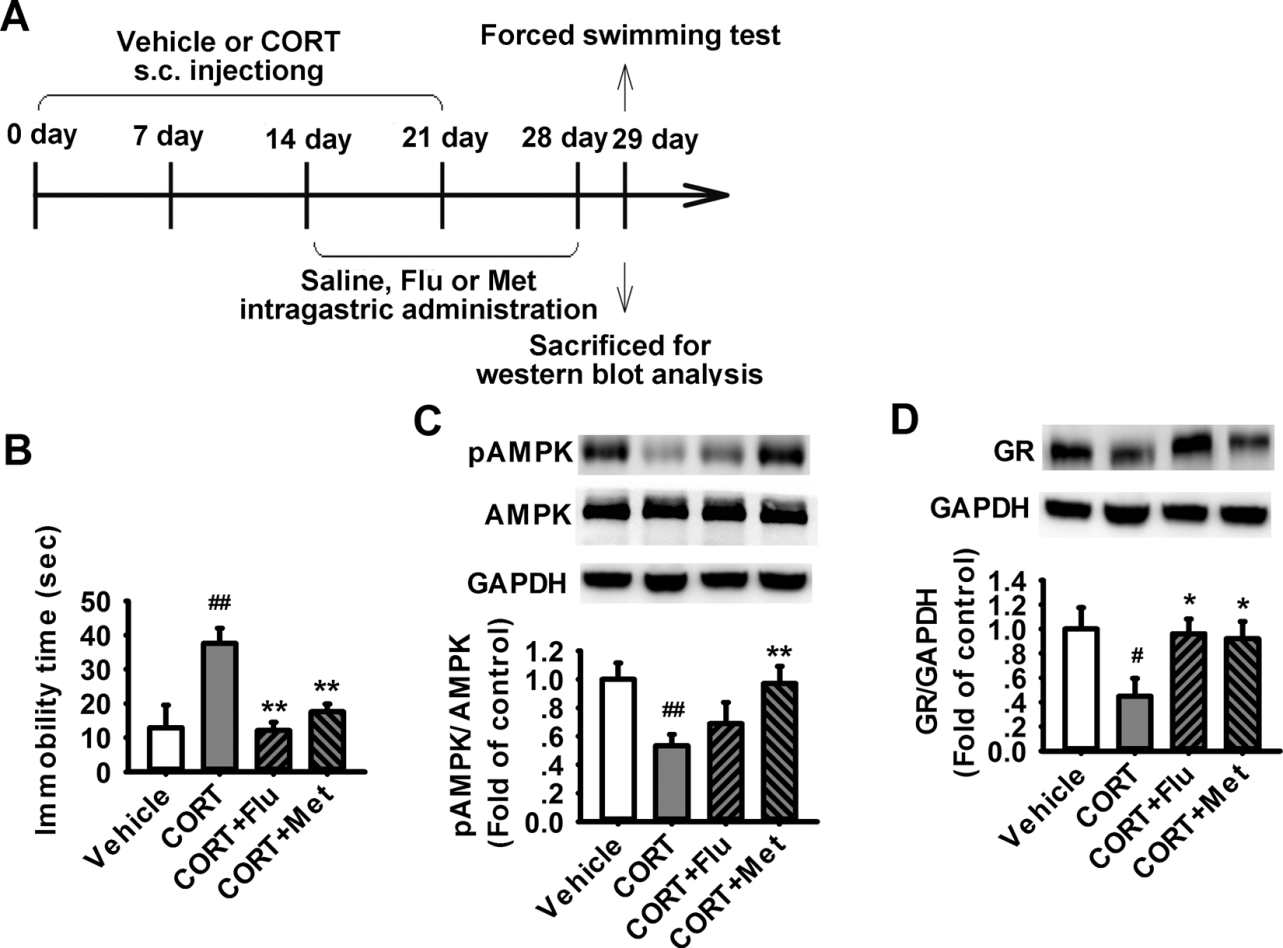
Effects of metformin on depressive behavior and expression of GR in prefrontal cortex of rat after chronic exposure to CORT. (A) Paradigm showing the procedure for administration of drugs and behavioral experiments. (B) Bar graph showing the immobility time of rats in the forced swimming test (FST). Treatment with metformin for 14 days significantly decreased the immobility time when compared to CORT group (CORT for 21 days) (n = 10–13 for each group). (C) Representative western blot images and summarized data showing the pAMPK (n = 10 for each group) (C) and GR (n = 7 for each group) (D) in PFC from the rats treated with vehicle, CORT, CORT+Flu or CORT+Met. *P < 0.05, **P < 0.01 vs vehicle. ^#^P < 0.05, ^##^P < 0.01 vs CORT.

We then examined the effects of AMPK activator metformin (300 mg/kg, intragastric administration, 14 days) on chronic CORT exposure (40 mg/kg, subcutaneous injection, 21 days)-induced behavior alteration and downregulation of GR in prefrontal cortex of rats (Fig 6A). As shown in Fig 6, the immobility time in the CORT exposure group was significantly longer than that in the control group. Meanwhile, both metformin and fluoxetine efficiently attenuated the increase of immobility time of the rats subjected to CORT exposure (Fig 6B). We also examined the alterations of GR and pAMPK in rat prefrontal cortex. The results showed that chronic exposure of CORT led to downregulations of pAMPK (Fig 6C) and GR (Fig 6D), which were significantly prevented by the treatments with fluoxetine or metformin (Fig 6C and 6D). However, treatment with metformin or fluoxetine had no effect on the loss of body weight induced by CORT exposure in rats (S2 Fig). These results suggest metformin improves the depression-like behavior induced by chronic exposure to CORT. Metformin also rescues the decreased levels of pAMPK and GR induced by CORT in the prefrontal cortex (PFC) of rats.

## Discussion

In the present study, we investigated the mechanism by which GCs inhibits the expression of GR in cultured prefrontal cortical astrocytes. We found that chronic exposure to excessive GCs led to decreased expression of GR in rat prefrontal cortical astrocytes, which was associated with the reduced level of AMPK phosphorylation. Activation of AMPK prevented DEX induced downregulation of GR and depression-like behavior in rats. It was further shown that DEX promoted the phosphorylation of FOXO3a and suppressed the expression of LKB1, which were mediated by SGK1 as SGK1 inhibitor attenuated the effects of DEX. Furthermore, AMPK inactivation-induced downregulation of GR in prefrontal cortical astrocytes is mediated by the dephosphorylation of HDAC5. Together, our data support the hypothesis that AMPK is crucial in modulating the expression of GR in rat prefrontal cortical astrocytes in chronic stress.

Reduced number and altered morphology of glial cells in PFC have been shown to be involved in the pathophysiology of MDD [19, 21, 32, 33]. Astrocytes act as an important information barrier and transporter between the central nervous system (CNS) and the periphery [34, 35]. In the chronic stress, the release of neurotrophic factors from astrocytes is reduced, which leads to the structural changes and even apoptosis of the neuronal cells [18]. It has also been shown that the stimulation of endogenous ATP release from astrocytes could induce antidepressant-like effects in mouse models of depression [6], suggesting that astrocyte-derived ATP may be an important modulator in the regulation of depressive behaviors. These studies suggest that astrocytes play important roles in depression. Therefore, we investigated the functional alterations of astrocytes in the PFC under the condition of chronic GC stress, to determine the intrinsic involvement of astrocytes in chronic stress-induced disorders. In agreement with previous studies [22, 36], we demonstrated that excessive and chronic exposure of GCs reduced the expression of GR mainly in rat prefrontal cortical astrocytes, but not in neurons, suggesting that chronic exposure of GCs maybe mainly affected the level pAMPK and GR in astrocytes, however it could not exclude the possibility of effects on other cells. In addition, we further showed that the decreased expression of GR was associated with decreased phosphorylation of AMPK, suggesting that AMPK may contribute to the downregulation of GR in response to GCs stress. Indeed, administration of AMPK inhibitors or down-regulation of the AMPK expression level by knockdown of AMPKα subunit reduced the expression of GR, and AMPK agonists reversed the decreased expression of GR induced by chronic exposure to GCs. These results clearly demonstrated that decreased phosphorylation of AMPK is the cause of down-regulated GR, which was further proved by the fact that GR inhibitor RU38486 could not prevent the decreased phosphorylation of AMPK induced by DEX. When the GR was blocked, the regulation of pAMPK by DEX seems was more pronounced. The reason may be that the activity of GR mediated by GCs can promote the phosphorylation of AMPK depending on the concentration and duration of GCs, and GR blocker can prevent this effect [37].

Our results also indicated the ineffectiveness of GR inhibitor in preventing DEX-induced effects, which raises the possibility that DEX might act on other targets in addition to GR. Indeed, the previous study has shown that GCs not only promote the expression of SGK1, but also directly activate SGK1 [38]. SGK1 is an immediate-early gene responding to GCs [39, 40]. SGK1 activity is not merely a downstream event of GR activation, but also involved in regulating CORT-induced nuclear translocation of GR [30]. GR-dependent upregulation of SGK1 may be a supporting mechanism for maintaining and prolonging GR activation after exposure to increased GC levels, such as stress, and even after GC levels have returned to normal range [30, 31]. In line with these studies, our further exploration showed that blockade of SGK1 can prevent the decline of AMPK phosphorylation and GR expression induced by chronic exposure to excessive GCs, indicating that SGK1 activation mediates these changes induced by chronic GCs exposure.

AMPK acts as a sensor of cellular energy status, which is activated by the increase of cellular AMP:ATP ratio [27]. AMPK is allosterically activated by high AMP concentrations [41]. Once activated, AMPK switches on ATP-producing pathways and simultaneously inhibits ATP-consuming processes in order to sustain energy homeostasis [42]. The activation of AMPK in response to elevated AMP involves phosphorylation by an upstream kinase LKB1. Thus, we examined the effect of DEX on LKB1 expression. Consistent with the decreased phosphorylation of AMPK, our results revealed that chronic exposure to GCs decreased the expression of LKB1. Furthermore, as a GR-targeting transcription-associative factor, FOXO3 coordinates the expression of LKB1 and other target genes depending on the metabolic status of the cell [31, 43, 44]. Translocation of FOXO3a into nucleus promotes the transcription of the LKB1, while GCs-induced activation of SGK1 phosphorylates FOXO3a and inhibits its transcriptional activity [31]. Consistently, we showed that DEX significantly increased the phosphorylation of FOXO3a at Thr32 sites in a SGK1-dependent manner. Taken together, our results demonstrated that GC-induced downregulation of GR is mediated by the SGK1-FOXO3a-LKB1-AMPK signaling pathway.

After establishing the upstream signaling pathway of AMPK, we continued to delineate downstream signaling pathway of AMPK mediating the downregulation of GR by chronic GCs exposure. It has been demonstrated that activation of AMPK inhibits the nuclear translocation and the deacetylation activity of HDAC5. HDAC5 is the main histone deacetylase in brain, and it contributes to the transcription of a series of related genes via phosphorylation at two serine residues-Ser498 and Ser259 [26, 28]. First of all, we examined the effect of DEX on HDAC5 phosphorylation. Our results clearly showed that chronic DEX exposure decreased the phosphorylation of HDAC5, which could be prevented by the application of AMPK activator AICAR. It therefore suggests that HDAC5 plays a role in the downregulation of GR by DEX treatment. More importantly, HDAC5 inhibitors SAHA and SB939 both effectively blocked DEX-induced downregulation of GR in cultured prefrontal cortical astrocytes. These results indicate that HDAC5 is involved in AMPK-mediated downregulation of GR.

Chronic stress can be practically mimicked by chronic subcutaneous injection of corticosterone in the stress model of rats [4, 45]. We found that AMPK agonist metformin, as well as fluoxetine decreased the immobility time of rats exposed to chronic corticosterone exposure in forced swimming test. This finding suggests that adequate activation of AMPK has antidepressant effects, although the underlying molecular mechanisms need further elucidation.

In conclusion, we initiatively assumed and demonstrated the hypothesis that the functional declines of AMPK and GR in prefrontal cortical astrocytes are intimately associated with the process of chronic GCs exposure, which may play critical roles in the chronic stress-induced pathogenesis of depression. We testified that the reduction in activated AMPK mediated the decreased expression of GR induced by chronic exposure to GCs, via at least partially HDAC5 signaling pathway. Our findings indicate that the AMPK activator metformin may represent a potential candidate for preventive and therapeutic intervention against chronic stress-induced psychiatric disorders, such as depression.

## Supporting Information

S1 Fig (PDF)

S2 Fig (PDF)

## References

[1] Law YW, Yip PS, Zhang Y, Caine ED. The chronic impact of work on suicides and under-utilization of psychiatric and psychosocial services. J Affect Disord. 2014;168: 254–261. 10.1016/j.jad.2014.06.031 25064810 PMC4180047

[2] Meaney MJ, Diorio J, Francis D, Widdowson J, LaPlante P, Caldji C, et al Early environmental regulation of forebrain glucocorticoid receptor gene expression: implications for adrenocortical responses to stress. Dev Neurosci. 1996;18(1–2): 49–72. 8840086 10.1159/000111395

[3] Karanth S, Linthorst AC, Stalla GK, Barden N, Holsboer F, Reul JM. Hypothalamic-pituitary-adrenocortical axis changes in a transgenic mouse with impaired glucocorticoid receptor function. Endocrinology. 1997;138(8): 3476–3485. 9231802 10.1210/endo.138.8.5331

[4] Johnson SA, Fournier NM, Kalynchuk LE. Effect of different doses of corticosterone on depression-like behavior and HPA axis responses to a novel stressor. Behav Brain Res. 2006;168(2): 280–288. 16386319 10.1016/j.bbr.2005.11.019

[5] Lussier AL, Lebedeva K, Fenton EY, Guskjolen A, Caruncho HJ, Kalynchuk LE. The progressive development of depression-like behavior in corticosterone-treated rats is paralleled by slowed granule cell maturation and decreased reelin expression in the adult dentate gyrus. Neuropharmacology. 2013;71: 174–183. 10.1016/j.neuropharm.2013.04.012 23608736

[6] Cao X, Li LP, Wang Q, Wu Q, Hu HH, Zhang M, et al Astrocyte-derived ATP modulates depressive-like behaviors. Nat Med. 2013;19(6): 773–777. 10.1038/nm.3162 23644515

[7] Iio W, Takagi H, Ogawa Y, Tsukahara T, Chohnan S, Toyoda A. Effects of chronic social defeat stress on peripheral leptin and its hypothalamic actions. BMC Neurosci. 2014;15: 72 10.1186/1471-2202-15-72 24906408 PMC4059170

[8] Zhu S, Wang J, Zhang Y, Li V, Kong J, He J, et al Unpredictable chronic mild stress induces anxiety and depression-like behaviors and inactivates AMP-activated protein kinase in mice. Brain Res. 2014;1576: 81–90. 10.1016/j.brainres.2014.06.002 24971831

[9] Kahn BB, Alquier T, Carling D, Hardie DG. AMP-activated protein kinase: ancient energy gauge provides clues to modern understanding of metabolism. Cell Metab. 2005;1(1): 15–25. 16054041 10.1016/j.cmet.2004.12.003

[10] Kola B, Boscaro M, Rutter GA, Grossman AB, Korbonits M. Expanding role of AMPK in endocrinology. Trends Endocrinol Metab. 2006;17(5): 205–215. 16766204 10.1016/j.tem.2006.05.006

[11] Lim CT, Kola B, Korbonits M. AMPK as a mediator of hormonal signalling. J Mol Endocrinol. 2010;44(2): 87–97. 10.1677/JME-09-0063 19625456

[12] Liu F, Benashski SE, Persky R, Xu Y, Li J, McCullough LD. Age-related changes in AMP-activated protein kinase after stroke. Age (Dordr). 2012;34(1): 157–168. 21360073 10.1007/s11357-011-9214-8 PMC3260368

[13] Minokoshi Y, Alquier T, Furukawa N, Kim YB, Lee A, Xue B, et al AMP-kinase regulates food intake by responding to hormonal and nutrient signals in the hypothalamus. Nature. 2004;428(6982): 569–574. 15058305 10.1038/nature02440

[14] Yi CO, Jeon BT, Shin HJ, Jeong EA, Chang KC, Lee JE, et al Resveratrol activates AMPK and suppresses LPS-induced NF-kappaB-dependent COX-2 activation in RAW 264.7 macrophage cells. Anat Cell Biol. 2011;44(3): 194–203. 10.5115/acb.2011.44.3.194 22025971 PMC3195823

[15] Kola B, Christ-Crain M, Lolli F, Arnaldi G, Giacchetti G, Boscaro M, et al Changes in adenosine 5'-monophosphate-activated protein kinase as a mechanism of visceral obesity in Cushing's syndrome. J Clin Endocrinol Metab. 2008;93(12): 4969–4973. 10.1210/jc.2008-1297 18782871 PMC7611639

[16] Puthanveetil P, Rodrigues B. Glucocorticoid excess induces accumulation of cardiac glycogen and triglyceride: suggested role for AMPK. Curr Pharm Des. 2013;19(27): 4818–4830. 23323614 10.2174/13816128113199990340

[17] Nader N, Ng SS, Lambrou GI, Pervanidou P, Wang Y, Chrousos GP, et al AMPK regulates metabolic actions of glucocorticoids by phosphorylating the glucocorticoid receptor through p38 MAPK. Mol Endocrinol. 2010;24(9): 1748–1764. 10.1210/me.2010-0192 20660302 PMC2940476

[18] Takano K, Yamasaki H, Kawabe K, Moriyama M, Nakamura Y. Imipramine induces brain-derived neurotrophic factor mRNA expression in cultured astrocytes. J Pharmacol Sci. 2012;120(3): 176–186. 23076128 10.1254/jphs.12039fp

[19] Banasr M, Duman RS. Glial loss in the prefrontal cortex is sufficient to induce depressive-like behaviors. Biol Psychiatry. 2008;64(10): 863–870. 10.1016/j.biopsych.2008.06.008 18639237 PMC2709733

[20] Xia M, Abazyan S, Jouroukhin Y, Pletnikov M. Behavioral sequelae of astrocyte dysfunction: focus on animal models of schizophrenia. Schizophr Res. 2014. 10.1016/j.schres.2014.10.044 PMC4439390 25468180

[21] Cotter D, Mackay D, Chana G, Beasley C, Landau S, Everall IP. Reduced neuronal size and glial cell density in area 9 of the dorsolateral prefrontal cortex in subjects with major depressive disorder. Cereb Cortex. 2002;12(4): 386–394. 11884354 10.1093/cercor/12.4.386

[22] Unemura K, Kume T, Kondo M, Maeda Y, Izumi Y, Akaike A. Glucocorticoids decrease astrocyte numbers by reducing glucocorticoid receptor expression in vitro and in vivo. J Pharmacol Sci. 2012;119(1): 30–39. 22641130 10.1254/jphs.12047fp

[23] Liu J, Wang F, Huang C, Long LH, Wu WN, Cai F, et al Activation of phosphatidylinositol-linked novel D1 dopamine receptor contributes to the calcium mobilization in cultured rat prefrontal cortical astrocytes. Cell Mol Neurobiol. 2009;29(3): 317–328. 10.1007/s10571-008-9323-9 18975071 PMC11505845

[24] Wu WN, Wu PF, Zhou J, Guan XL, Zhang Z, Yang YJ, et al Orexin-A activates hypothalamic AMP-activated protein kinase signaling through a Ca(2)(+)-dependent mechanism involving voltage-gated L-type calcium channel. Mol Pharmacol. 2013;84(6): 876–887. 10.1124/mol.113.086744 24068427

[25] Borsoi M, Antonio CB, Viana AF, Nardin P, Goncalves CA, Rates SM. Immobility behavior during the forced swim test correlates with BNDF levels in the frontal cortex, but not with cognitive impairments. Physiol Behav. 2015;140: 79–88. 10.1016/j.physbeh.2014.12.024 25496978

[26] McGee SL, Hargreaves M. AMPK and transcriptional regulation. Front Biosci. 2008;13: 3022–3033. 17981775 10.2741/2907

[27] Spasic MR, Callaerts P, Norga KK. AMP-activated protein kinase (AMPK) molecular crossroad for metabolic control and survival of neurons. Neuroscientist. 2009;15(4): 309–316. 10.1177/1073858408327805 19359670

[28] Zhao JX, Yue WF, Zhu MJ, Du M. AMP-activated protein kinase regulates beta-catenin transcription via histone deacetylase 5. J Biol Chem. 2011;286(18): 16426–16434. 10.1074/jbc.M110.199372 21454484 PMC3091248

[29] Moguilewsky M, Philibert D. RU 38486: potent antiglucocorticoid activity correlated with strong binding to the cytosolic glucocorticoid receptor followed by an impaired activation. J Steroid Biochem. 1984;20(1): 271–276. 6708512 10.1016/0022-4731(84)90216-4

[30] Anacker C, Cattaneo A, Musaelyan K, Zunszain PA, Horowitz M, Molteni R, et al Role for the kinase SGK1 in stress, depression, and glucocorticoid effects on hippocampal neurogenesis. Proc Natl Acad Sci U S A. 2013;110(21): 8708–8713. 10.1073/pnas.1300886110 23650397 PMC3666742

[31] Lutzner N, Kalbacher H, Krones-Herzig A, Rosl F. FOXO3 is a glucocorticoid receptor target and regulates LKB1 and its own expression based on cellular AMP levels via a positive autoregulatory loop. PLoS One. 2012;7(7): e42166 10.1371/journal.pone.0042166 22848740 PMC3407083

[32] Ongur D, Drevets WC, Price JL. Glial reduction in the subgenual prefrontal cortex in mood disorders. Proc Natl Acad Sci U S A. 1998;95(22): 13290–13295. 9789081 10.1073/pnas.95.22.13290 PMC23786

[33] Banasr M, Chowdhury GM, Terwilliger R, Newton SS, Duman RS, Behar KL, et al Glial pathology in an animal model of depression: reversal of stress-induced cellular, metabolic and behavioral deficits by the glutamate-modulating drug riluzole. Mol Psychiatry. 2010;15(5): 501–511. 10.1038/mp.2008.106 18825147 PMC3347761

[34] Cabezas R, Avila M, Gonzalez J, El-Bacha RS, Baez E, Garcia-Segura LM, et al Astrocytic modulation of blood brain barrier: perspectives on Parkinson's disease. Front Cell Neurosci. 2014;8: 211 10.3389/fncel.2014.00211 25136294 PMC4120694

[35] Perea G, Sur M, Araque A. Neuron-glia networks: integral gear of brain function. Front Cell Neurosci. 2014;8: 378 10.3389/fncel.2014.00378 25414643 PMC4222327

[36] Gadek-Michalska A, Spyrka J, Rachwalska P, Tadeusz J, Bugajski J. Influence of chronic stress on brain corticosteroid receptors and HPA axis activity. Pharmacol Rep. 2013;65(5): 1163–1175. 24399712 10.1016/s1734-1140(13)71474-9

[37] Liu L, Song Z, Jiao H, Lin H. Glucocorticoids increase NPY gene expression via hypothalamic AMPK signaling in broiler chicks. Endocrinology. 2014;155(6): 2190–2198. 10.1210/en.2013-1632 24693963

[38] Webster MK, Goya L, Ge Y, Maiyar AC, Firestone GL. Characterization of sgk, a novel member of the serine/threonine protein kinase gene family which is transcriptionally induced by glucocorticoids and serum. Mol Cell Biol. 1993;13(4): 2031–2040. 8455596 10.1128/mcb.13.4.2031-2040.1993 PMC359524

[39] Anacker C, Zunszain PA, Cattaneo A, Carvalho LA, Garabedian MJ, Thuret S, et al Antidepressants increase human hippocampal neurogenesis by activating the glucocorticoid receptor. Mol Psychiatry. 2011;16(7): 738–750. 10.1038/mp.2011.26 21483429 PMC3121947

[40] Sato H, Horikawa Y, Iizuka K, Sakurai N, Tanaka T, Shihara N, et al Large-scale analysis of glucocorticoid target genes in rat hypothalamus. J Neurochem. 2008;106(2): 805–814. 10.1111/j.1471-4159.2008.05489.x 18489715

[41] Oakhill JS, Chen ZP, Scott JW, Steel R, Castelli LA, Ling N, et al beta-Subunit myristoylation is the gatekeeper for initiating metabolic stress sensing by AMP-activated protein kinase (AMPK). Proc Natl Acad Sci U S A. 2010;107(45): 19237–19241. 10.1073/pnas.1009705107 20974912 PMC2984171

[42] Viollet B, Andreelli F, Jorgensen SB, Perrin C, Flamez D, Mu J, et al Physiological role of AMP-activated protein kinase (AMPK): insights from knockout mouse models. Biochem Soc Trans. 2003;31(Pt 1): 216–219. 12546688 10.1042/bst0310216

[43] Pew T, Zou M, Brickley DR, Conzen SD. Glucocorticoid (GC)-mediated down-regulation of urokinase plasminogen activator expression via the serum and GC regulated kinase-1/forkhead box O3a pathway. Endocrinology. 2008;149(5): 2637–2645. 10.1210/en.2007-1096 18239069 PMC2329267

[44] Wu W, Zou M, Brickley DR, Pew T, Conzen SD. Glucocorticoid receptor activation signals through forkhead transcription factor 3a in breast cancer cells. Mol Endocrinol. 2006;20(10): 2304–2314. 16690749 10.1210/me.2006-0131

[45] Seib LM, Wellman CL. Daily injections alter spine density in rat medial prefrontal cortex. Neurosci Lett. 2003;337(1): 29–32. 12524164 10.1016/s0304-3940(02)01287-9

