# Supplementary material for: AMPK Mediates Glucocorticoids Stress-Induced Downregulation of the Glucocorticoid Receptor in Cultured Rat Prefrontal Cortical Astrocytes
Source: PLoS One. 2016 Aug 11;11(8):e0159513. doi: 10.1371/journal.pone.0159513 (PMC4981361; doi:10.1371/journal.pone.0159513)
Supplement: S1 Fig — (PDF) [file pone.0159513.s001.pdf]

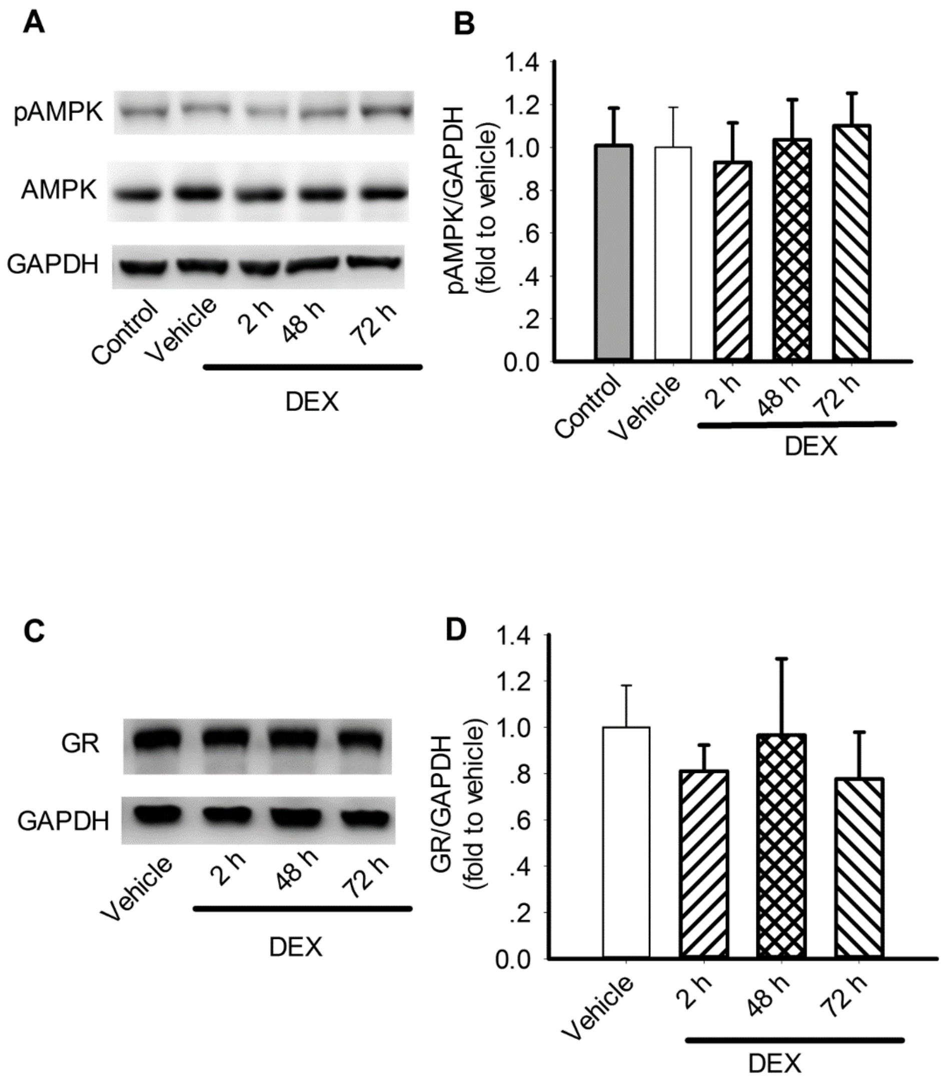

S1 Fig. Exposure to glucocorticoids does not affect the levels of pAMPK and GR in cultured rat prefrontal cortical neurons. Representative western blot (A) and summarized data (B) showing the level of pAMPK in cultured neurons exposed to DEX for 2 h, 48 h and 72 h. (n = 5 for each group). Representative western blot (C) and summarized data (D) showing the expression of GR in cultured neurons exposed to DEX for 2 h, 48 h and 72 h. (n = 5 for each group).
