# Supplementary material for: AMPK Mediates Glucocorticoids Stress-Induced Downregulation of the Glucocorticoid Receptor in Cultured Rat Prefrontal Cortical Astrocytes
Source: PLoS One. 2016 Aug 11;11(8):e0159513. doi: 10.1371/journal.pone.0159513 (PMC4981361; doi:10.1371/journal.pone.0159513)
Supplement: S2 Fig — (PDF) [file pone.0159513.s002.pdf]

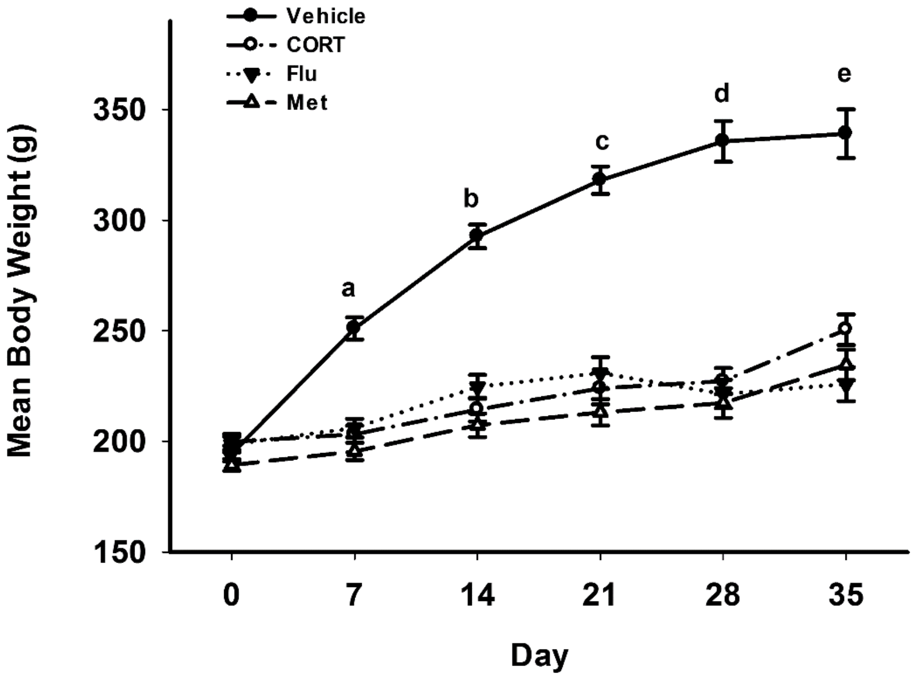

S2 Fig. Treatment with metformin or fluoxetine had no effect on the loss of body weight induced by CORT exposure in rats. Treatment with metformin for 14 days had no effect on the loss of body weight compared to CORT group (CORT for 21 days) (n = 10-13 for each group). The rats were weighted every day during administration. Mean body weight of Met group does not differ with that in CORT group, as well as Flu group. Data represent mean ± S.E.M.. a, b, c, d, e, $P < 0.01$, vs CORT group.
